# Impact of Antioxidant Feed and Growth Manipulation on the Redox Regulation of Atlantic Salmon Smolts

**DOI:** 10.3390/antiox11091708

**Published:** 2022-08-30

**Authors:** Peng Yin, Björn Thrandur Björnsson, Per Gunnar Fjelldal, Takaya Saito, Sofie Charlotte Remø, Rolf Brudvik Edvardsen, Tom Hansen, Sandeep Sharma, Rolf Erik Olsen, Kristin Hamre

**Affiliations:** 1 Institute of Marine Research, 5817 Bergen, Norway; 2 Department of Biological Sciences, University of Bergen, 5020 Bergen, Norway; 3 Department of Biological and Environmental Sciences, University of Gothenburg, 411 24 Gothenburg, Sweden; 4 Institute of Marine Research, Matre Aquaculture Research Station, 5984 Matredal, Norway; 5 Biomar AS, 7010 Trondheim, Norway; 6 Institutt for Biologi Fakultet for Naturvitenskap, Norwegian University of Science and Technology, 7491 Trondheim, Norway

**Keywords:** redox, transcriptional regulation, fish, antioxidant nutrients, growth hormone, oxidative stress

## Abstract

Accumulating evidence indicates a close relationship between oxidative stress and growth rate in fish. However, the underlying mechanisms of this relationship remain unclear. This study evaluated the combined effect of dietary antioxidants and growth hormone (GH) on the liver and the muscle redox status of Atlantic salmon. There were two sequential experimental phases (EP) termed EP1 and EP2, each lasting for 6 weeks. In EP1, Atlantic salmon were fed either low-(L, 230 mg/kg ascorbic acid (Asc), 120 mg/kg α-tocopherol (α-TOH)), or high-(H, 380 mg/kg Asc, 210 mg/kg α-TOH)vitamin diets. The vitamins were supplemented as stable forms and the feeding was continued in EP2. In EP2, half of the fish were implanted with 3 μL per g body weight of recombinant bovine GH (Posilac^®^, 1 mg rbGH g BW^−1^) suspended in sesame oil, while the other half were held in different tanks and sham-implanted with similar volumes of the sesame oil vehicle. Here, we show that increasing high levels of vitamin C and E (diet H) increased their content in muscle and liver during EP1. GH implantation decreased vitamin C and E levels in both liver and muscle but increased malondialdehyde (MDA) levels only in the liver. GH also affected many genes and pathways of antioxidant enzymes and the redox balance. Among the most consistent were the upregulation of genes coding for the NADPH oxidase family (NOXs) and downregulation of the oxidative stress response transcription factor, nuclear factor-erythroid 2-related factor 2 (nrf2), and its downstream target genes in the liver. We verified that GH increases the growth rate until the end of the trail and induces an oxidative effect in the liver and muscle of Atlantic salmon. Dietary antioxidants do lower oxidative stress but have no effect on the growth rate. The present study is intended as a starting point to understand the potential interactions between growth and redox signaling in fish.

## 1. Introduction

The Atlantic salmon is the most important aquaculture species in Norway. The post-smolts are farmed in open sea cages and their growth rates are affected by environmental factors; for example, the seasonal variations, especially the changes in daylength and temperature [1,2,3]. A close relationship exists between oxidative stress and growth rates in Atlantic salmon. Antioxidants, such as vitamin C, E, or astaxanthin are reduced in the tissues of Norwegian-farmed Atlantic salmon during the fast growth period in spring concomitant with increases in oxidation products such as thiobarbituric acid reactive substances (TBARS), one of which is malondialdehyde (MDA) [4,5,6], an end product of lipid peroxidation. The levels of those antioxidants are often restored in the late summer and the fish develop severe cataracts during this period [6]. Cataracts are a well-known effect of oxidative stress to the lens [7]. Furthermore, growth hormone transgenic fish that have a high growth rate are known to have elevated oxidative stress [8,9,10,11]. These studies indicate a possible involvement of redox regulation. However, the underlying mechanisms of the relationship between redox regulation and growth remain unclear.

ROS are highly oxidative, and the uncontrolled accumulation is toxic, which can damage biological molecules such as proteins, lipids, and nucleic acids, and consequently result in oxidative stress associated with certain diseases [12,13]. Beyond the role of reactive oxygen species (ROS) as damage signals, recent research has established that redox signaling regulates metabolism and several cellular processes [14]. ROS include superoxide anions (O_2_^−^) and hydrogen peroxide (H_2_O_2_), where H_2_O_2_ is a result of the dismutation of O_2_^−^ by superoxide dismutase (Sod). O_2_^−^ is produced mainly by NADPH oxidases [15,16] and the mitochondrial electron transport chain (ETC) among other enzymatic generators [17]. At low physiological levels, H_2_O_2_ is the major agent in redox signaling through reversible oxidation reactions of thiol groups in proteins, thereby affecting protein function, and changing signal output, enzyme activity, and gene transcription [18,19]. For example, particular redox-sensitive cysteines in Kelch-like ECH-associated protein 1 (Keap1) are modified by oxidants such as H_2_O_2_. This leads to a conformational change of Keap1, which prevents nuclear factor-erythroid 2-related factor 2 (Nrf2) ubiquitylation. This allows Nrf2 to accumulate in the nucleus and induces the transcription of target genes. The target genes code for proteins involved in maintaining the redox balance and/or are involved in redox signaling. Examples are thioredoxin (Txn) and the glutathione (GSH) synthesis/recycling genes, and genes coding for ROS and xenobiotic detoxification [20]. On the other hand, increased oxidative stress may reduce the glutathione/glutathione disulfide and cysteine/cystine ratios and significantly affect the cell redox status. For example, the intracellular redox environment is generally more reduced in cell proliferation, while more oxidative in differentiation and even more in apoptosis [21]. Control of the redox environment is essential for proper development in fish as in other vertebrates [22,23,24,25].

Cells have therefore developed intricate antioxidant systems consisting of enzymes, endogenously synthesized antioxidants, and antioxidant nutrients to control the redox status. Briefly, there is a collection of fast-acting antioxidant enzymes that can neutralize the ROS, such as superoxide dismutase (Sod), glutathione peroxidase (Gpx), and catalase (Cat) [26]. Sod dismutates O_2_^−^ into H_2_O_2_ and O_2_, Cat and Gpx neutralizes H_2_O_2_, producing H_2_O and O_2_. GSH, thioredoxins, and glutaredoxins reduce most of the oxidatively modified residues and maintains the balance of the thiol-related redox status [21,27]. In addition, antioxidant nutrients such as vitamin C and E are organic compounds necessary for the antioxidant system and must be supplied in appropriate amounts in the diet of fish [28,29]. Vitamin E is recycled by ascorbate [30], constituting an antioxidant network in interaction with thiol redox cycles [31]. 

Growth hormone (GH) is the principal regulator of vertebrate growth that stimulates growth independently or in conjunction with insulin-like growth factor I (IGF-I) [32,33]. Several GH transgenic fish show dramatically enhanced growth rates under specific experimental conditions [11,34,35]. Growing evidence indicates that ROS are important for growth signaling. In plants, ROS act as a second messenger mediating growth signals of hormones [36]. In animals, many growth factor receptors are targets of ROS, and the protein kinases such c-Jun N-terminal kinase and p38 mitogen-activated protein kinase, which mediate cellular processes including proliferation, differentiation, and apoptosis, are regulated by oxidants [37]. Further, oxidative stress (H_2_O_2_) has been demonstrated to affect the IGF-I signaling pathway [38]. Considering the important role of redox signaling in the regulation of pathways, we hypothesize that ROS, probably H_2_O_2_ [39], may serve as a second messenger for growth signaling, and thereby contribute to the growth-promoting effects induced by GH. To test this, we fed salmon different levels of dietary antioxidant nutrients and implanted fish with GH to investigate whether and how the redox system changed. We analyzed endogenous and nutritional antioxidants, antioxidant enzymes, and the redox system transcriptome in the muscle and liver of Atlantic salmon based on their importance within the redox system and/or because of their close relationship with the redox system [40,41,42]. 

## 2. Materials and Methods

### 2.1. Fish and Research Ethics 

Atlantic salmon (Salmo salar) of strain AquaGen Atlantic QTL-innOva IPN from AquaGen AS (Trondheim, Norway) were used in the current experiment. The parr were reared on an off-season 0+ age smoltification protocol that included rearing under constant light (LD 24:0) until 11 July 2019 when an almost 8-week photoperiod “winter” was introduced with a LD12:12. After being returned on LD24:0 on 2 September, smoltification was induced, after which the fish were transferred to seawater (26 g salts L^−1^) as smolts on 1 October.

The study was carried out in compliance with the Norwegian Animal Welfare Act guidelines and the regulations concerning experiments with living animals were approved by the Norwegian Food Safety Authority (FOTS ID 21500). The fish trial was conducted at the Institute of Marine Research (Matre, Matredal, 61° N, Western Norway).

### 2.2. Diets

Two experimental diets were produced by Biomar AS, Tech Centre (Brande, Denmark). Both diets contained the same basal mixture of ingredients including 25% fish meal, 11.75% fish oil, with the remaining plant ingredients (Table 1). The total protein and lipid content of the diets were 47% and 20%, respectively. The basal diet (low, L) contained 120 mg kg^−1^ tocopherol acetate (α-TOH) and 230 mg kg^−1^ ascorbyl biphosphate (Asc) while the high-vitamin diet (H) was supplemented with an additional 0.045% α-TOH and 0.035% Asc giving a total of 210 mg kg^−1^ α-TOH and 380 mg kg^−1^ Asc.

### 2.3. Experimental Design

On 14 August 2019, 360 Atlantic salmon parr were anesthetized (Finquel and bicarbonate, 0.1 g L^−1^, respectively) and PIT-tagged in the abdominal cavity with a 2 mm scalpel cut (Glass tag 2, 12 mm, TrackID AS, Stavanger, Norway). The fish were allowed to recover from the tagging and acclimate to in the new tanks for 3 weeks before the experiment started. The fish were randomly distributed into twelve tanks (water dept 0.4 m, 360 L) with 30 fish per tank, and fed a commercial feed (Skretting AS, Stavanger, Norway) during the acclimation period. The water temperature was kept constant at 12 °C throughout the experiment and the oxygen saturation in effluent water was always above 80%. 

**Figure 1:**
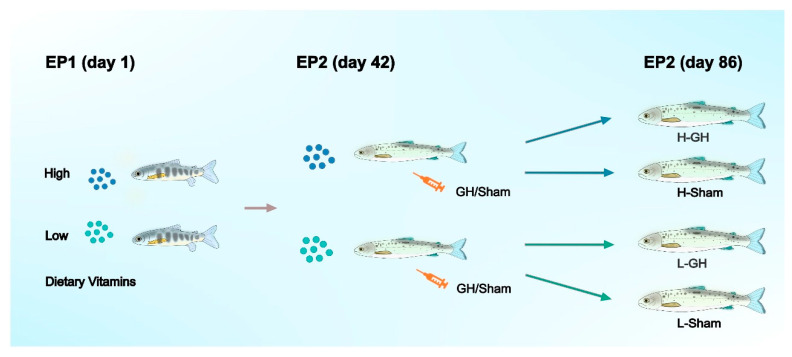
General flow chart of the experimental design of Atlantic salmon during EP1 and EP2.

The experimental design was presented in Figure 1. The feeding trial (experimental phase; EP1) commenced on 2 September (day 1). The photoperiod was changed to LD24:0 at this timepoint to induce smoltification. Salinity of the water was changed to 26 g L^−1^ on 1 October. Each tank contained 30 fish with mean initial body weight (BW) of 38.7 ± 0.6 g. The fish of six tanks were assigned to diet L, the other six tanks to diet H. The fish were fed continuously (24 h) and ad libitum. 

Experimental phase 2 (EP2): followed directly after EP1 and the feeding of the H and L diets continued. At the beginning of the EP2 (14 October) all fish were anaesthetized and 10 fish per tank sampled for analysis as detailed below. Of the remaining fish, 10 fish per tank were anesthetized and implanted intraperitoneally with growth hormone (GH) suspended in sesame oil (3 H- and 3 L-tanks) and 10 fish were injected with sesame oil only (Sham; 3 H- and 3 L-tanks). They were then distributed into separate tanks giving a 2 × 2 factorial design (H-GH, H-Sham, L-GH, L-Sham; see Figure 1). An approximately 5 mm incision was made in the abdominal wall and the implants were delivered through the incision, using a 250 μL positive-displacement pipette (Microman; Gilson, Middleton, WI, USA). Each fish received 3 μL g BW^−1^ of GH or sesame seed oil vehicle. The GH implant used was a sustained-release recombinant bovine GH (rbGH) formula (Posilac^®^, Monsanto Co., St Louis, MO, USA), resulting in a dose of 1 mg rbGH g BW^−1^ for the GH-treated fish. Following the procedure, the fish were allowed to recover from the anesthesia in aerated seawater from the facility before being returned to their respective tanks. No mortalities were noted. 

### 2.4. Sampling Procedure

Sampling was carried out on 14 October (day 42; end of EP1/start of EP2), 1 November (day 60) and 27 November (day 86; end of EP2). In the first sampling (42 days, end of EP1), all experimental fish were anesthetized and then BW was recorded. Ten fish per tank from the two dietary treatments in triplicate tanks (total sixty fish) were killed by an overdose of MS222 for analyses. On 1 November, only bulk weights of fish were recorded for minimum disturbance of the fish. In the last sampling (86 days, end of EP2), 120 fish (10 fish per tank, 12 tanks) were euthanized and then dissected for analyses after cataract evaluation and BW had been recorded. 

Lenses were carefully dissected from ten fish from each tank, put into separate tubes, immediately frozen on dry ice, and stored at −80 °C until analyses. Livers and muscle from three fish per tank were pooled for vitamin C and E analyses and frozen on dry ice. Individual samples of liver and muscle were dissected and flash frozen in liquid nitrogen until further analyses. The samples were transported on dry ice to the laboratory and stored at −80 °C until analyses.

### 2.5. Cataract Assessment 

Cataract assessment was performed according to the methods described [43,44]. Briefly, both lenses of 10 fish per tank were tested with a Kowa SL-15 slit-lamp microscope (Kowa, Tokyo, Japan) under darkened conditions. Each assessment was evaluated by the same person. Each fish lens was given a score of 0 to 4 according to its opacity, where 0 is no opacity and 4 is maximum opacity. The scores given are the sum from both eyes (0–8) [44]. 

### 2.6. Chemical Analyses of Diets and Organs

L-histidine (HIS) and Na-Acetyl-L-histidine (NAH) concentrations in individual lenses were determined by reversed-phase HPLC (Waters Corporation, Milford, MA, USA) [45], as modified by Breck et al. [46]. Supernatants for total (GSH) and oxidized (GSSG) glutathione were prepared from samples using a commercial kit (Prod. No. GT40, Oxford Biomedical Research, Oxford, UK) before being analyzed at 405 nm in a microplate reader (iEMS Reader Ms; Labsystems, Finland) [25]. Vitamin C was analyzed by HPLC as described previously [47] and tocopherols according to the CEN method [48]. Malondialdehyde (MDA) of liver and muscle were analyzed according to the method of Hamre et al. [6].

### 2.7. Antioxidant Enzyme Measurement

The activities of Sod, Gpx, Cat, glutathione reductase (Gr), and glutathione S-transferase (Gst) were analyzed with commercial kits (Items 706002 (Sod), 703102 (Gpx), 703202 (Gr), 703302 (Gst), 707002 (Cat); Cayman Chemical Co., Ann Arbor, MI, USA). Frozen tissues were homogenized (30 shakes per second for one and a half minutes) with ice cold-buffer (20 mM HEPES, 1mM EGTA, 90 mM mannitol and 70 mM sucrose, pH 7.2) in a ball mill (Retsch MM301 ball mill; Haan, Germany). For analyzing total Sod, the homogenized samples were centrifuged at 1500× *g* for 5 min at 4 °C, and the supernatant was collected. For analyses of Gpx, Gr, Gst, and Cat, the supernatants were further centrifuged at 10,000× *g* for 15 min at 4 °C and collected. The extracts were immediately stored at −80 °C for a maximum of 1 month before analyses. Total protein concentrations of supernatants were measured by a commercial assay kit (Prod. No. 22662, Thermo Scientific, Rockford, IL, USA). All enzyme activities were quantified on a microplate reader (iEMS Reader Ms; Labsystems, Finland) measuring Sod at 450 nm, Gpx, Gr, and Gst at 340 nm, Cat at 531 nm, and protein at 660 nm.

### 2.8. RNA Extraction and RNA Sequencing 

Total RNA of samples was prepared by using Promega simplyRNA kit (AX2420, Madison, WI, USA) and the Biomek 4000 automated workstation following the manufacturer’s instruction. The quality and quantity of RNA were measured by a Nanodrop spectrophotometer (ND-1000, NanoDrop Technologies, Wilmington, DE, USA) and all samples were evaluated for quality of RNA on the Agilent RNA 6000 Nano Kit in 2100 Bioanalyser (Agilent Technologies, Santa Clara, CA, USA). The isolated RNA was normalized to 50 μL/ng for RNA-sequencing by adding RNA-free double-distilled water (dd H_2_O). RNA-Seq library preparation was performed using the Illumina TruSeq Stranded mRNA library prep kit, according to the manufacturer’s protocol. Libraries were barcoded using unique barcodes and multiplexed before sequencing at the Illumina HiSeq4000 system. Library prep and sequencing were performed by the Genomics Core Facility (GCF) at the University of Bergen. 

The genome sequences (ICSASG_v2; Accession: GCA_000233375.4) and the RefSeq gene annotation (Annotation release ID: 100) of Atlantic salmon were downloaded from the National Center for Biotechnology Information (NCBI) website (https://www.ncbi.nlm.nih.gov/assembly/GCF_000233375.1; accessed on 30 July 2020). The target genes used in the present study were expanded corresponding *Atlantic salmon* genes by performing manual searches for matched gene symbols and gene names in RefSeq. A total of 316 genes involved in the redox system, covering antioxidant, thiol oxidoreductase, GSH synthesis/recycling, redox system transcription factors, NADPH oxidase family (NOXs), heat shock protein (HSP) and aquaporins (AQPs), DNA damage and repair, apoptosis, and inflammation were selected for analyses (see Supplementary Material for the full list of analyses). 

After the initial read quality checks by FastQC (https://qubeshub.org/resources/fastqc; accessed on 19 December 2019), adapters, short reads (less than 20 bases), and low-quality reads (phred score < Q30) were eliminated by Cutadapt [49] through Trim Galore (https://www.bioinformatics.babraham.ac.uk/projects/trim_galore/; accessed on 18 May 2018). Quality control reports were subsequently generated by MultiQC [50] for manual inspection. Prior to the alignment, the chromosome sequences were indexed by STAR [51] with the exon/intron information. All the reads that passed the quality control were aligned to the Atlantic salmon genome by STAR with the default parameters for paired-ends reads. Only uniquely aligned reads on the genome were selected for the subsequent quantification, which were counted to quantify gene expression by featureCount [52] with the default parameters for paired-ends reads. Only uniquely mapped reads to RefSeq genes were used for counting as the counts of unmapped, multi-mapped, and ambiguously mapped reads were discarded. The pipelines of the preprocessing as well as the following deferential gene expression analyses were developed and maintained using Snakemake [53]. 

Differentially expressed genes (DEGs) were calculated in a pairwise manner between six treatment groups of EP1 (H vs. L) and EP2 (H vs. L, H-GH vs. L-GH, H-GH vs. H-Sham, L-GH vs. L-Sham) by DESeq2 [54]. *p*-values of the fold changes between two groups were calculated by the Wald test from DESeq2 followed by multiple testing correction by the Benjamini–Hochberg procedure. The heatmaps were generated by ComplexHeatmap [55] to investigate the expression patterns of the target genes among different groups. 

### 2.9. Calculations

Specific growth rate (SGR, %) was calculated as: (lnBW*_f_* − lnBW*_i_*) × 100/number of feeding days, where BW*_i_* and BW*_f_* are the initial and final body weights (tank means), respectively.

Redox potential (Eh) is the half-cell potential for oxidation of GSH to GSSG and was calculated according to the Nernst equation:

Eh = E0′-RT/nF ln([GSH]^2^/[GSSG])

where R is the gas constant (R = 8.314 J K^−1^ mol^−1^), T the temperature (in Kelvin), and F the Faraday constant (F = 9.6485 × 10^4^ C mol^−1^). The units of GSH and GSSG concentrations, and Eh, are in moles and Volts, respectively. E0′ is the standard reduction potential and was assumed to be −240 mV at the environmental condition of 25 °C and pH 7 [21,56]. The measurements are calculated as the average of whole organs and do not take into account that the reduction potential varies between cell types and between organelles within the cells. 

### 2.10. Statistical Analyses 

Tanks (n = 3) were used as experimental unit for data on SGR (WP1, n = 6; WP2, n = 3) and vitamin C, vitamin E, and micro-mineral concentrations (n = 3). Individual fish was the experimental unit for data on cataract assessment (n = 30), on GSH and enzyme analyses (n = 9), on RNA-seq (n = 9) and on Lens HIS and NAH levels (n = 9). Data were analysed using Free software environment R version 4.0.4 (R Foundation for Statistical Computing, Vienna, Austria). Data from the first sampling of the two dietary treatments were subjected to the independent *T*-test. A 2 × 2-way factorial ANOVA was used to analyze the relationship between feed type and growth hormone implantation from the second sampling. Tukey’s HSD post-hoc test was used when interaction effects were found. Homogeneity of variances and normality were checked using Levene’s test and Shapiro–Wilk’s test, respectively. Data from EP1 and EP2 that were non-normal or heteroscedastic or both were analyzed using the Wilcox test and Scheirer–Ray–Hare extension of the Kruskal–Wallis test, respectively. The data of antioxidant enzyme activities were examined by principal component analysis PCA (R: factoextra, version 1.0.6) to explain the effects of GH or diet on antioxidant capacity of liver and muscle. Results are presented as means with standard deviation (mean ± SD) and a significance level of 95% was used (*p* < 0.05). Potential outliers were analyzed (ROUT, Q = 1) using GraphPad Prism, version 8 (GraphPad Software, La Jolla, CA, USA). Figures were made using GraphPad Prism. Differentially expressed genes (DEGs) of RNA-Seq data were defined when the genes had adjusted *p*-values < 0.1.

## 3. Results

### 3.1. Specific Growth Rate and Lens Health

**Figure 2:**
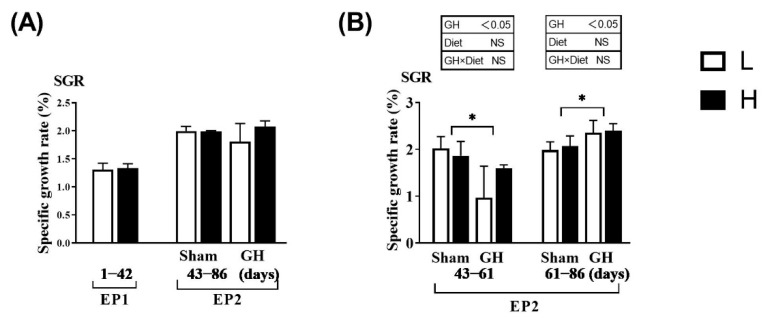
Specific growth rate in Atlantic salmon in response to dietary vitamins and growth hormone (GH) implantation or sham implantation over EP1 and EP2. The specific growth rate is calculated over the EP1 from 0 to 42 days, EP2 from 42 to 60 days as well as 61 to 86 days. Data presented as means ± SD. *p*-values obtained from two-way ANOVA on the main effects of diet, GH, and interaction are provided in insets. * Significant effect of treatment (*p* < 0.05, two-way ANOVA).

**Figure 3:**
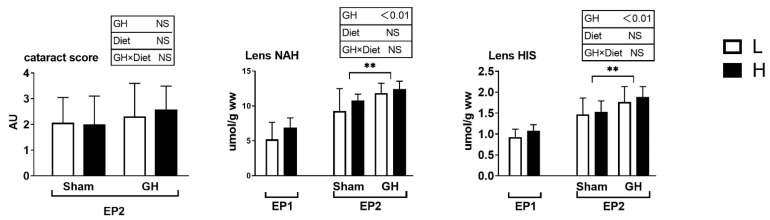
Cataract score, lens histidine, and lens N-acetyl L-histidine (NAH) in Atlantic salmon. Cataract score was only detected in the EP2. Data presented as means ± SD. *p*-values obtained from two-way ANOVA on the main effects of diet, GH, and interaction are provided in insets. **; significant effect of treatment (*p* < 0.01, two-way ANOVA).

The SGR did not vary between the L and H groups during EP1 and EP2 (*p* > 0.05; Figure 2A). Over the course of EP2, GH implantation initially reduced SGR (days 43–60, *p* > 0.05) compared with Sham, after which, the SGR increased until the end of the trial at 86 days (*p* < 0.05; Figure 2B). Overall, for days 43–86, there were no differences in growth rates between the groups. Neither GH nor diet affected the cataract score during EP2 (*p* > 0.05). There were significant increases in HIS and NAH in the lens from fish implanted with GH (*p* < 0.01; Figure 3).

### 3.2. Vitamin C, Vitamin E, and MDA Levels

**Figure 4:**
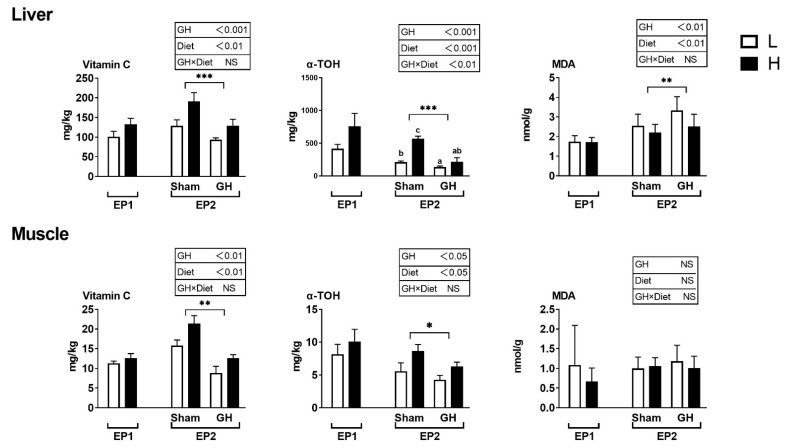
Tissue concentrations (mg kg^−1^ wet weight) of redox-dependent micronutrients in Atlantic salmon in response to dietary vitamins and GH implantation or sham implantation over EP1 and EP2. Data presented as means ± SD. *P*-values obtained from two-way ANOVA on the main effects of diet, GH, and interaction are provided in insets. *, **, ***; significant effect of treatment (*p* < 0.05, *p* < 0.01, *p* < 0.001, two-way ANOVA). Small letters are the result of post-hoc after significant interaction (two-way ANOVA with Tukey HSD post-hoc).

Vitamin C and E levels in tissues were not affected by the diets during EP1 (Figure 4). During EP2, both GH and diet effects were significant for vitamin C and E levels of both liver and muscle. Fish implanted with GH had generally reduced levels of these vitamins. There was a significant interaction between liver vitamin E (*p* < 0.05) and the H sham group, which resulted in the highest levels of vitamin E compared with other groups. Liver and muscle MDA were not affected by diet in the two groups in EP1 (*p* > 0.05). In EP2, liver MDA was affected by both dietary treatment and GH (*p* < 0.01), where GH increased MDA levels and high vitamin levels (diet H) reduced it. Muscle MDA was not affected by any treatment (*p* > 0.05). 

### 3.3. Glutathione Levels and Antioxidant Enzyme Activities

**Figure 5:**
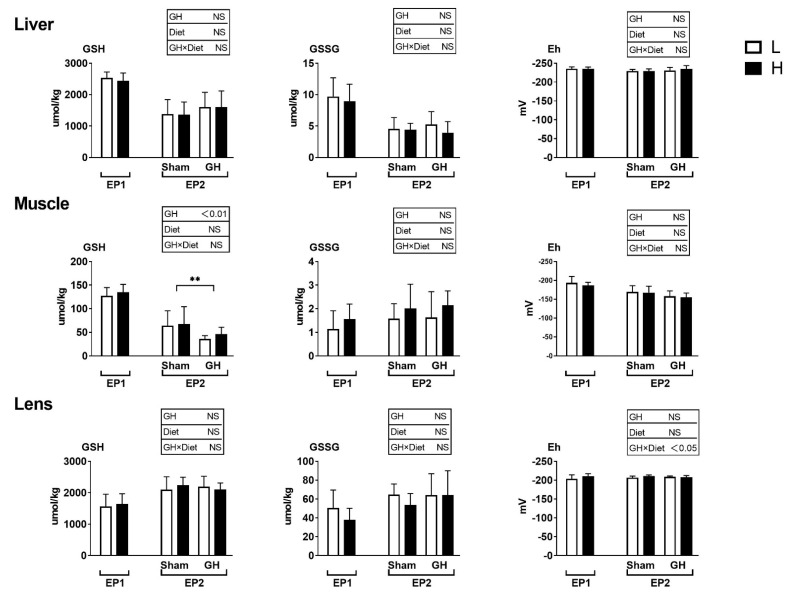
Tissues reduced and oxidized glutathione (GSH and GSSG, μmoles kg^−1^ wet weight) and the GSH-based redox potential (mV) in Atlantic salmon in response to dietary vitamins and GH implantation or sham implantation over EP1 and EP2. Data presented as means ± SD. *p*-values obtained from two-way ANOVA on the main effects of diet, GH, and interaction are provided in insets. **; significant effect of treatment (*p* < 0.01, two-way ANOVA).

The glutathione levels and redox potential in liver, muscle, and lens were not influenced by dietary treatment during EP1 (*p* > 0.05; Figure 5). GH reduced the GSH level in muscle but not in liver or lens during EP2. Further, there were no differences among treatment groups in muscle and liver redox potential during EP2. 

**Figure 6:**
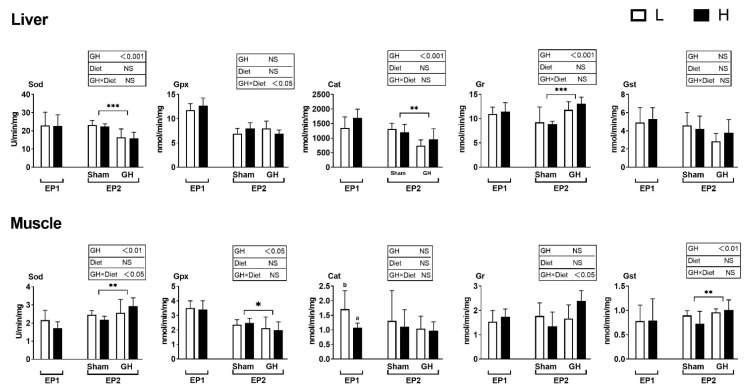
Changes in specific antioxidant enzyme activity in response to dietary vitamins and GH implantation or shame implantation over EP1 and EP2. Data presented as means ± SD. *P*-values obtained from two-way ANOVA on the main effects of diet, GH, and interaction are provided in insets. *, **, ***; significant effect of treatment (*p* ≤ 0.05, *p* ≤ 0.01, *p* ≤ 0.001, two-way ANOVA). Different l letters denote significant effect between groups in the EP1, small letters are the result of post-hoc after significant interactions (two-way ANOVA with Tukey HSD post-hoc) in the EP2.

**Figure 7:**
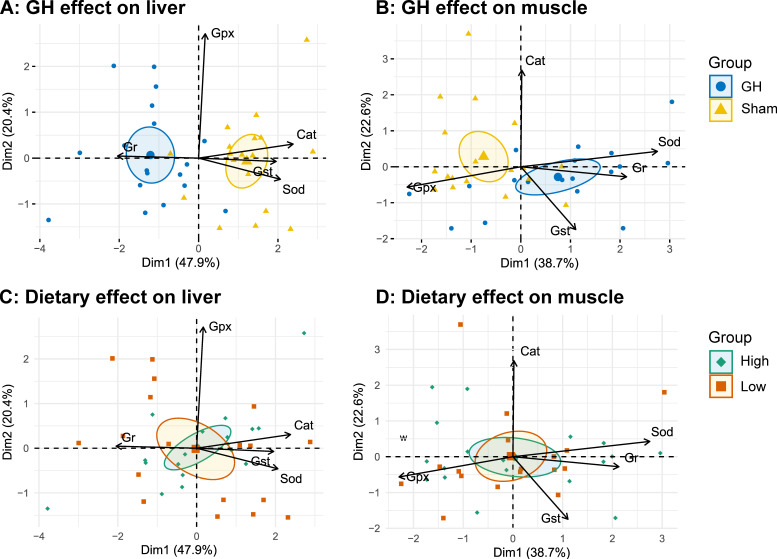
PCA biplots on specific antioxidant enzyme activity grouped by GH (**A**,**B**) or dietary vitamins C and E feeding (**C**,**D**) measured in liver and muscle, respectively. Arrows represent the 5 most contributing variables to the model, respectively (Cat, catalase; Sod, superoxide dismutase; Gst, glutathione S-transferase; Gpx, total glutathione peroxidase). Ellipses represent the 95% confidence intervals around a center of 9 rearing tanks (pool of 3 individuals).

In general, diet had little effect on the analyzed enzymes during EP1, except that fish fed diet H had a lower Cat activity compared to those fed L (*p* < 0.05, Figure 6). The PCA of antioxidant enzymes in liver and muscle during EP2 were grouped by GH (Figure 7A,B), but not by dietary vitamins C and E (Figure 7C,D). Accordingly, GH treatment significantly reduced Sod and Cat activities (*p* < 0.001) and increased the activity of Gr in liver (*p* < 0.05). There was a significant interaction for Gpx and GH (*p* < 0.05) where fish fed H tended to increase Gpx activity in the sham groups (H-Sham), while being reduced in the GH group (H-GH). GH on the other hand increased muscle Sod and Gst activities compared to the sham groups (*p* < 0.01) but reduced Gpx (*p* < 0.01). There were significant interactions for muscle Sod and diet (*p* < 0.01) where Sham-treated fish fed H (H-Sham) had reduced activity while the highest activity was found in the H-GH group. 

### 3.4. Mineral Concentrations

Levels of the redox-active metals Fe, Cu, Mn, Zn, and Se were measured in EP2 (Table 2). The levels of Se in liver and muscle were significantly decreased by GH (*p* < 0.05). GH increased Mn levels in liver (*p* < 0.05) but deceased them in muscle (*p* < 0.001). Fish fed the H had significantly higher Mn levels in muscle (*p* < 0.05).

### 3.5. Transcriptomic Assay and Gene Expression

**Figure 8:**
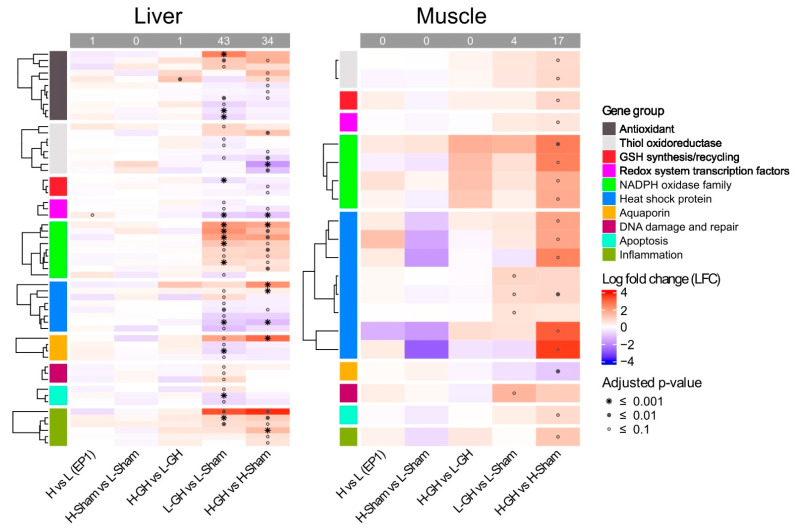
Expression of genes involved in redox system affected by dietary vitamins and GH implantation. Heat map of the expression of genes associated with the redox system over EP1 and EP2. Normalization genes was performed using size factors calculated by DESeq2, followed by log2 fold-change (LFC) and adjusted *p*-value estimates between sample group; red and blue indicate upregulated and downregulated expression, respectively, while the symbol *, •, and ⸰ indicate *p*-values lower than 0.001, 0.01, and 0.1, respectively.

In the EP1, diet had a minor effect on the regulation of redox-related genes. The only significantly differentially expressed gene found was *pgc-1a*, which was upregulated in H-fed fish. In the EP2, H upregulated only *ucp2* compared to L among GH-treated groups (H-GH vs. L-GH). Both GH and diets induced differential regulation of the redox system (Figure 8). Here, GH significantly affected 43 genes in the liver of L-fed fish (L-GH vs. L-Sham) and 34 genes of H-fed fish (H-GH vs. H-Sham). In contrast, fewer genes were affected by GH in muscle, where 4 and 17 genes were in L-fed and H-fed fish, respectively.

The summary of the DEGs (L-GH vs. L-Sham, H-GH vs. H-Sham) and their proportions in the respective groups are shown in Table 3. The approximate proportions of DEGs of several biologically relevant groups are listed. In the liver, 21% (7 out of 33) and 18% (6 out of 33) of the genes coding for antioxidant enzymes in L- and H-fed fish, respectively, were DEGs. Of these, GH upregulated *gpx3* and *mta* and downregulated *mnsod*, *cat*, and *ucp2* in L-fed fish, and upregulated *gpx2* and *mta* while downregulated *mnsod* as well as *msrb2* in H-fed fish. Of the group of genes coding for thiol oxidoreductases and GSH synthesis/recycling, *txnrd1* was significantly upregulated and *txnl1*, *glrx*, *prxl2c*, and *gclr* gene expression were significantly downregulated in L-fed fish, while upregulated *txn* and downregulated *txn2, prxl2ctxinp*, and *gclc* were found in H-fed fish. In addition, GH downregulated liver gene expression of the *nrf2* and *pgc-1a* in both dietary groups. A total of 53% (8 out of 15) of the genes coding for NOXs were affected by GH in both dietary groups. GH significantly upregulated the expressions of *ncf1*, *ncf2, cyba, cybb*, and *spd2a* genes in both L- and H-fed fish, but the *nox1* gene was downregulated in L-fed fish, only, while *cyba* was upregulated in H-fed fish. The HSP genes were affected by GH. As well as upregulated *hopl*, GH upregulated *hsp70* and *hsp90b1* in L-fed and H-fed fish respectively, while GH downregulated *hsf1*, *dnajb4, hspa8, hspa9, hsp90b1*, and *hs90a* in L-fed fish, expression of *hsf1* was only downregulated inf H-fed fish.

Genes coding for AQPs and DNA damage and repair were more affected and apoptosis in L-fed fish compared to that in H-fed fish. Here, 57% (4 out of 7) of these genes were affected by GH where the *aqp9* homologs were upregulated or downregulated, and *aqp8* was downregulated in L-fed fish. Only *aqp9* was upregulated in fish fed the H. Twenty percent (3 out of 15) and twelve percent (3 out of 26) of genes involved in DNA damage and repair and apoptosis were affected by GH only in L-fed fish, respectively, e.g., *tp53*, *gadd45a*, *ogg1*, and *casp9* were upregulated and *bcl-2* and *bcl-xl* were downregulated. In contrast, GH affected more genes involved in inflammation in the H-fed fish compared to L-fed fish (46% vs. 25%). GH upregulated the *Ikba* and *il-8* in both diet groups, while *nf-kb* was upregulated only in the H-fed fish. 

In muscle, GH upregulated the expression of genes involved in thiol oxidoreductase, GSH synthesis/recycling, redox transcription factors, NOXs, HSP, apoptosis, and inflammation in H-fed fish (H-GH vs. H-Sham); *txnip*, *gr*, *nrf2*, *ncf1*, *ncf2*, *cybb*, *spd2a*, *hsp30*, *hspa9*, *casp9*, and *ikba*, and downregulated *aqp3*. The expression of genes involved in HSP and DNA damage and repair: *hsp60*, *hspa9*, and *gadd45a*, were upregulated in fish fed L (L-GH vs. L-Sham).

## 4. Discussion

The present study strengthens the notion that there is a functional relationship between GH and the oxidative stress of fish. Vitamin E is fat soluble and tissue concentrations in Atlantic salmon increase linearly with dietary inclusions [29]. Studies in mice indicate that damage to tissues and oxidative stress deplete vitamin C and E storages [57,58]. This was very much in line with our results where GH treatment led to reductions of vitamins C and E, which clearly suggest that the fish had elevated oxidative stress. This notion is strengthened by GH-stimulated increased liver accumulation of the lipid oxidation product MDA. Despite these changes in oxidative stress status, there were no changes in the redox potential. This clearly indicates that salmon are capable of controlling the redox environment under these conditions.

**Figure 9:**
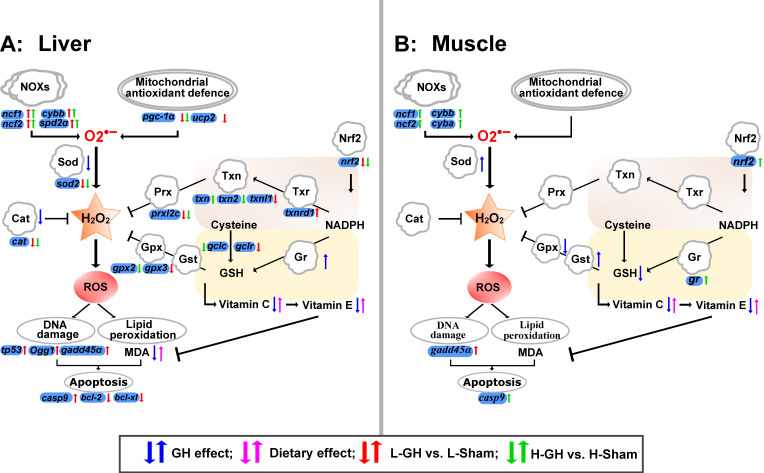
Effect of GH and vitamin C and E nutrition on the redox system in liver (**A**) and muscle (**B**) of Atlantic salmon during the EP2. The systemic overview of redox regulation was raised based on the results in this study. Arrows in blue color represent the regulation of antioxidants by GH, in pink color represent the regulation of antioxidants by dietary vitamin C and E. Arrows in red and green color represent the regulation of redox relative gene expression of the comparisons of L-GH vs. L-Sham and H-GH vs. H-Sham, respectively.

An interesting notion of the present study was that the GH effect on the antioxidant defense and transcription regulation of the redox system appeared to be regulated through Nrf2 modulation. Pgc-1 α protein is an important regulator of mitochondrial function and homeostasis, regulating the expression of mitochondrial antioxidant genes and thus preventing oxidative stress and mitochondrial dysfunction [59]. The loss of *nrf2* and *pgc-1a* genes cripples the antioxidant defense system and leads to increased oxidative stress [60]. The downregulation of *nrf2*, *pgc-1a*, and their cytoprotective target genes such as *gclc*, *gclr*, *sod*, and *cat* indicate that GH suppresses antioxidant activity in the cell and in mitochondria of the liver, respectively. Accordingly, GH decreased the enzyme activities of both Sod and Cat in the liver. Microminerals such as Mn, Se, Fe, Cu, or Zn are closely linked to the redox system of Atlantic salmon [61], where Se is an essential component of the selenoproteins such as Gpx, and Mn is a required component of Mnsod (Sod2) for reducing mitochondrial oxidative stress [62]. Besides the critical role of Mn in managing the superoxide level, oxidative stress is induced when Mn is excessive [63]. The present study demonstrates that decreased Se levels in GH-treated fish are highly correlated with suppression of the antioxidant system, while the liver Mn levels were negatively correlated with Sod activity and *mnsod* expression. Similarly, hepatic Mnsod activity has been found to be reduced in fish that had a high level of Mn [64,65]. Based on the results and the redox-active role of Se and Mn, GH affected the Se and Mn levels, which may impact the redox environment of the tissues of Atlantic salmon. NOXs can be activated by various growth factors and cytokines, exemplified by IGF-I [66]. The upregulation of NOX genes corresponds with higher ROS generation [8,67,68,69]. In the present study, the upregulation of liver genes such as *ncf1*, *ncf2*, and *cybb* coding for essential components of NOXs suggests that GH might promote ROS generation by upregulating NOXs expression. Taken together, it is reasonable to suggest that GH could have effects on ROS metabolism by affecting the sources of ROS and antioxidative defenses in the liver of Atlantic salmon. We raised a systemic overview of redox regulation in the GH-treated Atlantic salmon liver and muscle (Figure 9).

Our results suggest that the effects of GH on the muscle of Atlantic salmon are tissue-specific, and these interact with dietary antioxidants. In the muscle of H-fed fish, more redox genes were affected by GH than in L-fed fish, including *nrf2* and NOX genes, which were both upregulated. Nrf2 signaling has been shown to be upregulated by the antioxidant vitamins [70,71,72] and to interact with the expression of NOXs [73,74]. It is therefore possible that H_2_O_2_ production by NOX signaling was increased by GH and that Nrf2 provided protection in the muscle, and that the H diet might promote a healthier growth signal than the L diet. However, of greater importance may be that increased Sod activity increases the dismutation of O_2_^−^ to H_2_O_2_, and without simultaneous increases in the activities of H_2_O_2_-reducing enzymes, such as Gpx, which will lead to the accumulation of H_2_O_2_. Hormones that regulate the growth and defense pathway in plants use ROS as second messengers [75]. If GH promotes growth through ROS as second messengers in animals, a possible outcome by increasing dietary antioxidant vitamins is growth inhibition. This cannot be deduced from the results of the present study. However, the trial period was short and the variation in growth was too large to be able to see such effects. 

In the current study, the levels of vitamin E and vitamin C applied in the L and H diets are both above the suggested requirements at 150 mg kg^−1^ of a-TOH and 190 mg kg^−1^ of Asc, respectively, given in a previous study of Atlantic salmon [5]. However, increased liver MDA levels were found in L-fed fish during the EP2. GH also has effects on DNA damage and repair signaling pathways [76,77], which are suggested to be involved in redox regulation [78,79]. Here, the upregulated expression of *tp53*, *gadd45a*, *casp9*, and lowered mRNA levels of anti-apoptosis genes including *bcl2* and *bcl-xl* indicates elevated DNA damage and apoptosis in the liver of L-fed fish. These data suggest that diet L may have insufficient vitamin C and E levels to protect fish from oxidative stress when GH levels are high. 

Oxidative stress is considered a major risk factor for cataract development [80]. The occurrence of cataracts coincided with increased growth and was accompanied by the alteration of the redox environment of lenses in a recent study on Atlantic salmon [6]. NAH is proposed as an osmolyte [81] as well as an antioxidant [82] in Atlantic salmon. The concentrations of NAH and HIS are dependent on dietary HIS [83], and a low level of NAH is consequently regarded as a marker for the risk of cataract development. Here, GH did not affect cataracts, but did affect the cataract-related parameters. GSH-based Eh indicates that the lens was slightly more oxidized in fish implanted with GH and the high NAH and HIS indicate that GH increased antioxidant defense in salmon lenses. 

In summary, high GH levels lead to increased oxidation in Atlantic salmon, as seen by the decrease in tissue vitamin C and E levels, higher MDA, regulation of the activity of antioxidant enzymes, and GSH. It appears likely that GH induces redox signaling derived from NOXs signaling, mitochondrial ROS production, and from modulation of the Nrf2 pathway to weaken the ROS sinks. These processes are affected by dietary antioxidants. The L diet, which is close to the recommended levels of vitamin C and E, seems to give insufficient protection against oxidation and the H diet seems to promote healthy growth in response to high levels of GH. This indicates that GH involves redox signaling and may use ROS as second messengers.

## Figures and Tables

**Table 1:** Ingredient and chemical composition of the experimental diets.

Ingredients (%)	Diet L	Diet H
Fish Meal LT ^1^	12.5	12.5
Fish Meal NA ^2^	12.5	12.5
Soy protein concentrate ^3^	14.89	14.89
Pea protein concentrate ^4^	15	15
Wheat gluten ^5^	6.6	6.6
Guar meal ^6^	5.4	5.4
Wheat meal ^7^	12.2	12.12
Fish oil ^8^	11.75	11.75
Rapeseed oil ^9^	5.04	5.04
Monocalcium phosphate	3.97	3.97
Amino acid mix	0.63	0.63
Premix vitamins, minerals, and others	1.11	1.11
Vitamin E (%) added ^10^	0	0.035
Vitamin C (%) added ^11^	0	0.045
Analytical composition (% dw)		
Protein	47	47
Lipid	20	20
Ash	7.7	8.0
Dry matter	94	95
Micronutrients involved in redox regulation (mg kg^−1^ dw)		
Ascorbic acid	230	380
a-TOH	120	210
γ-TOH	21	21

^1.^ Nordsilmel, Norway. ^2.^ 999, Denmark. ^3.^ Koster, Brazil. ^4.^ Promill, China. ^5.^ Roquette, Germany. _6_. Sunita, India. ^7.^ Hedegaard, Denmark. ^8.^ Koster, Peru. ^9.^ Scanola Denmark. ^10.^ Miavit, Germany (50%). ^11.^ Hebei Tianyin, China (35%).

**Table 2:** Micro-mineral concentration in the liver and muscle of Atlantic salmon during EP2 (mg/kg wet weight.

	Sham		GH		*p* Value		
	L-Sham	H-Sham	L-GH	H-GH	Diet	GH	Diet × GH
Liver							
Se	1.33 ± 0.21	1.30 ± 0.10	1.07 ± 0.14	1.09 ± 0.11	NS	<0.05	NS
Mn	1.70 ± 0.17	1.75 ± 0.21	2.20 ± 0.35	1.87 ± 0.06	NS	<0.05	NS
Fe	70.33 ± 23.46	71.00 ± 51.29	57.67 ± 24.01	41.67 ± 13.58	NS	NS	NS
Cu	38.33 ± 4.51	34.33 ± 5.03	32.67 ± 10.69	34.67 ± 11.55	NS	NS	NS
Zn	23.00 ± 1.00	22.00 ± 1.00	23.33 ± 1.15	24.00 ± 2.00	NS	NS	NS
Muscle							
Se	0.18 ± 0.01	0.17 ± 0.01	0.16 ± 0.01	0.17 ± 0.01	NS	<0.05	NS
Mn	0.36 ± 0.04	0.23 ± 0.06	0.19 ± 0.02	0.14 ± 0.04	<0.05	<0.001	NS
Fe	1.53 ± 0.49	1.33 ± 0.06	1.37 ± 0.15	1.35 ± 0.07	NS	NS	NS
Cu	0.21 ± 0.02	0.22 ± 0.02	0.32 ± 0.20	0.21 ± 0.03	NS	NS	NS
Zn	4.53 ± 0.23	4.40 ± 0.10	4.47 ± 0.06	4.50 ± 0.14	NS	NS	NS

*p*-value indicates statistical significance as obtained through two-way ANOVA, n = 3. Se: selenium; Mn: manganese; Fe: iron; Cu: copper; Zn: zinc.

**Table 3:** A summary of the changes in differentially expressed genes (DEGs) involved in redox system regulation in response to GH and dietary vitamin C and E in liver and muscle of Atlantic salmon.

Tissue	L-GH vs. L-Sham			H-GH vs. H-Sham		
Liver	Affected (%) *	Upregulated ^†^	Downregulated ^†^	Affected (%) *	Upregulated ^†^	Downregulated ^†^
Antioxidant	(7/33) 21	*gpx3*, *mta*	*mnsod2*, *cat, ucp2*	(6/33) 18	*gpx2*, *mta*	*mnsod*, *msrb2*
Thiol oxidoreductase	(4/42) 10	*txnrd1*	*txnl1*, *glrx*, *prxl2c*	(5/41) 12	*txn*	*txn2*, *txnip*, *prxl2c*
GSH synthesis/recycling	(1/8) 13		*gclr*	(2/8) 25		*gclc*
NADPH oxidase system	(8/15) 53	*ncf1*, *ncf2*, *cybb*, *spd2a*	*nox1*	(8/15) 53	*ncf1*, *ncf2*, *cyba*, *cybb*, *spd2a*	
Redox system transcription factors	(3/8) 38		*nrf2*, *pgc-1α*	(2/9) 22		*nrf2*, *pgc-1α*
Heat shock protein	(7/52) 13	*hsp70, hopl*	*hsf1*, *dnajb4*, *hspa8*,	(4/52) 8	*hopl*, *hsp90b1*	*hsf1*
			*hspa9, hsp90b1*, *hs90a*			
Aquaporin	(4/7) 57	*aqp9*	*aqp8*, *aqp9*	(1/8) 13	*aqp9*	
DNA damage and repair	(3/15) 20	*tp53*, *gadd45a*, *ogg1*		(0/15) 0		
Apoptosis	(3/26) 12	*casp9*	*bcl2*, *bcl-xl*	(0/26) 0		
Inflammation	(3/12) 25	*ikbα*, *il-8*		(6/13) 46	*nf-kb*, *ikbα*, *il-8*	
Antioxidant	(0/31) 0			(0/31) 0		
Thiol oxidoreductase	(0/41) 0			(2/40) 5	*txnip*	
GSH synthesis/recycling	(0/8) 0			(1/8) 13	*gr*	
NADPH oxidase system	(0/13) 0			(4/13) 31	*ncf1*, *ncf2*, *cyba*, *cybb*	
Redox system transcription factors	(0/7) 0			(1/7) 14	*nrf2*	
Heat shock protein	(3/62) 5	*hsp60, hspa9*		(6/62) 10	*hsp30*, *hspa9*	
Aquaporin	(0/4) 0			(1/4) 25		*aqp3*
DNA damage and repair	(1/15) 7	*gadd45a*		(0/15) 0		
Apoptosis	(0/25) 0			(1/25) 4	*casp9*	
Inflammation	(0/11) 0			(1/11) 9	*ikbα*	

* Differentially expressed genes/total mapped genes. ^†^ Some gene symbols are associated with multiple gene IDs. Gene abbreviation and name: *gpx2*: glutathione peroxidase 2; *gpx3*: glutathione peroxidase 3; *mnsod*: superoxide dismutase 2; *cat*: catalase; *ucp2*: uncoupling protein 2; *mta*: metallothionein A; *msrb2*: methionine sulfoxide reductase B2; *txn*: thioredoxin; *txnrd1*: thioredoxin reductase 1, cytoplasmic-like; txnl1: thioredoxin-like 1; *glrx*: glutaredoxin; *prxl2c*: peroxiredoxin-like 2C; *gclr*: glutamate--cysteine ligase regulatory subunit; *gclc*: glutamate–cysteine ligase, catalytic subunit; *gr*: glutathione reductase; *ncf1*: neutrophil cytosolic factor 1; *ncf2*: neutrophil cytosolic factor 2; *cyba*: cytochrome b-245 light chain; *cybb*: cytochrome b-245 heavy chain; *spd2a*: SH3 and PX domain-containing protein 2A; *nox1*: NADPH oxidase 1; *nrf2*: nuclear factor erythroid 2-related factor 2; *pgc-1α*: peroxisome proliferator-activated receptor gamma coactivator 1-alpha-like; *hsp70*: heat shock 70 kDa protein; *hopl*: hsp70-Hsp90 organizing protein-like; *hsf1*: heat shock transcription factor 1; *dnajb4*: DnaJ heat shock protein family (hsp40) member B4; *hsp60*: heat shock protein 60; *hsp70*: heat shock protein 70; *hspa8*: heat shock protein 8; *hsp90*: heat shock protein 90; *aqp3*: aquaporin 3; *aqp8*: aquaporin 8; *aqp9*: aquaporin 9; *tp53*: tumor protein p53; *gadd451*: growth arrest and DNA damage-inducible protein GADD45 alpha; *ogg1*: 8-oxoguanine DNA glycosylase; *casp9*: caspase 9; *bcl2*: apoptosis regulator Bcl-2-like; *bcl-xl*: Bcl-2-like1; *nf-kb*: transcription factor p65; *ikbα*: NF-kappa-B inhibitor alpha; *il-8*: interleukin 8.

## Data Availability

The authors declare that the data supporting the findings of this study are available within the article and its Supplementary Information. Raw RNA-seq data have been uploaded to the Sequence Read Archive (SRA) on the NCBI website and are available under BioProject PRJNA765504 and PRJNA765887.

## Supplementary Materials

The following supporting information can be downloaded at: https://www.mdpi.com/article/10.3390/antiox11091708/s1.
